# A New Strategy for the Treatment of Old Corrugated Container Pulping Wastewater by the Ozone-Catalyzed Polyurethane Sponge Biodegradation Process

**DOI:** 10.3390/polym16101329

**Published:** 2024-05-09

**Authors:** Yuxuan Cai, Shaozhe Huang, Jianhua Xiong

**Affiliations:** 1 School of Resources, Environment and Materials, Guangxi University, Nanning 530004, China; 2 Guangxi Key Laboratory of Emerging Contaminants Monitoring, Early Warning and Environmental Health Risk Assessment, Nanning 530004, China

**Keywords:** polyurethane sponge, coupling, microenvironment, biodegradation

## Abstract

Old Corrugated Container (OCC) pulping wastewater has a complex organic composition and high levels of biotoxicity. The presence of dissolved and colloidal substances (DCSs) is a major limiting factor for pulp and paper companies to achieve closed-water recycling. In order to solve this problem, the coupled ozone-catalyzed oxidation and biodegradation (OCB) method was used to treat OCC pulping wastewater in this study. A polyurethane sponge was used as the basic skeleton, loaded with nano TiO_2_ and microorganisms, respectively, and then put into a reactor. After an 8-min ozone-catalyzed oxidation reaction, a 10-h biological reaction was carried out. The process was effective in removing organic pollutants such as COD and BOD_5_ from OCC paper whitewater. The removal rates of COD and BOD_5_ were 81.5% and 85.1%, respectively. By using the polyurethane sponge to construct a microenvironment suitable for microbial growth and metabolism, this study successfully applied and optimized engineered bacteria—white rut fungi (WRF)—in the system to achieve practical degradation of OCC pulping wastewater. Meanwhile, the biocompatibility of different microbial communities on the polyurethane sponge was analyzed by examining the degradation performance of OCC pulping wastewater. The structure of microbial communities loaded on the polyurethane sponge was analyzed to understand the degradation mechanism and microbial reaction behavior. White-rot fungi (*Phanerochaete*) contributed more to the degradation of OCC wastewater, and new strains adapted to OCC wastewater degradation were generated.

## 1. Introduction

The paper industry is a large consumer of water resources in the industrial category, and the current statistics show that each ton of paper generation consumes about 10 to 300 tons of water [1]. China’s pulp and paper industry is in a stage of rapid development, according to statistics. To date, more than 250 organic pollutants have been detected in paper wastewater [2] from the wood itself as well as from various papermaking auxiliaries.

With the rapid growth of the e-commerce industry in recent years, the consumption of corrugated boxes and boxboard has increased, with the production of corrugated board in 2023 being 29.74 million tons. Almost all raw materials for corrugated raw paper and containerboard production come from recycled used containerboard (OCC), and unlike traditional papermaking wastewater, OCC pulping wastewater is recycled multiple times and thus has a large number of fine adhesives in it, which are usually stabilized in the wastewater system in the form of dissolved and colloidal substances (DCSs) [3]. The sources of adhesives in OCC pulping wastewater are mainly adhesives in glues and printing inks, as well as resins in the waste pulp [4]. Therefore, when treating OCC wastewater, an effect evaluation is an important indicator in addition to conventional water quality indicators, and the content of adhesives is also an important indicator.

At present, the treatment methods for DCSs in OCC wastewater are mainly divided into physical, chemical, and biological methods. Physical methods apply simple and commonly used flocculation and filtration methods to remove adhesives; chemical methods include adding chemical additives for the adsorption or solidification of adhesives [5]; and biological methods are categorized into bio-enzymatic and bio-bacterial methods. The bio-enzymatic method is highly controllable and is more often applied to lipase and laccase [6] in paper wastewater treatment, but its application cost is higher; the bio-bacterial method mostly uses engineered bacteria like white-rut fungi (WRF). WRF can produce enzymes that effectively degrade cellulose substances present in large quantities in paper wastewater, such as manganese peroxidase and laccase; therefore, WRF are widely used in biological treatment [7].

Advanced oxidation processes have been applied to paper whitewater removal because of their good removal effect on difficult-to-degrade pollutants [8]. Ozone catalytic oxidation technology is a highly efficient technology for degrading organic pollutants that is gradually being paid attention to, and the removal of chromaticity and COD in the case of paper wastewater is remarkable. Researchers used a prepared catalyst for the ozone-catalyzed deep treatment of paper wastewater with a remarkable effect, reporting the color and COD removal rates of 58.5% and 77.5%, respectively [9]. In addition, ozone-catalyzed oxidation technology can improve the biochemistry of paper wastewater.

Individual ozone catalytic oxidation technology has the disadvantages of high ozone depletion, high reaction selectivity, low mineralization rate, etc., and the combination of ozone catalytic oxidation and other process technologies has become a hotspot of current research. Su [10] used ozone oxidation and biodegradation synergistic technology to treat simulated tetracycline hydrochloride wastewater and real coking wastewater biological effluent and found that the removal rate of tetracycline hydrochloride could reach 97%. In addition, the removal rate of COD in real coking wastewater increased to 34%, which increased the removal rate by 76%. Zhou [11] used microbubble ozonation catalytic oxidation and biochemical synergistic technology to treat real coal chemical wastewater deeply. He found that biological treatment alone can reduce COD by 40%, and the ozone-catalyzed oxidation–biochemical synergistic process can improve COD removal by 26.7% to reach 66.7%.

This research endeavor is engineered to address the treatment of Old Corrugated Container (OCC) pulping wastewater by leveraging an integrated approach that synergizes ozone-catalyzed oxidation with biodegradation, hereinafter referred to as the Ozone-Catalyzed Biodegradation (OCB) process. Central to this innovative treatment modality is the utilization of a polyurethane (PU) sponge, which serves as the foundational matrix within a bespoke reactor construct. The reactor is meticulously designed to incorporate nanoscale titanium dioxide (TiO_2_) and a consortium of microorganisms, which are co-localized within the porous architecture of the PU sponge. Following an initial phase of ozone-induced catalytic oxidation, the subsequent stage of the process engages the microorganisms to effectuate the biodegradation of the recalcitrant components within the OCC pulping wastewater. This sequential treatment strategy is anticipated to culminate in the efficient degradation of organic pollutants, thereby augmenting the overall efficacy of the wastewater remediation process. At the same time, microbial strains that are biocompatible with the polyurethane sponge were screened as the microbial system in the OCB. In addition, we treated OCC pulping wastewater using simple biodegradation (B) to compare the degradation effect of the OCB system. The degradation performance of the coupled system showed significantly higher COD and DCS removal compared with the B system. In addition, the structural changes in microorganisms were analyzed to explain the mechanism of microbial action on pollutants. The whole process is simple to operate and can be used for the deep treatment of OCC pulping wastewater. Therefore, the use of a polyurethane sponge loaded with specially engineered bacterial strains and catalytic ozonation for the treatment of OCC wastewater in this study can provide a reference for practical application.

## 2. Materials and Methods

### 2.1. Materials and Methods

All chemicals used in this experiment were of analytical grade (purity 98%) and required no further purification. The chemicals included magnesium sulfate heptahydrate (MgSO_4_·7H_2_O), calcium chloride (CaCl_2_), sodium dihydrogen phosphate dihydrate (NaH_2_PO_3_·2H_2_O), sodium dihydrogen phosphate dodecahydrate (Na_2_HPO_4_·12H_2_O), sulfuric acid (H_2_SO_4_), silver sulfate (Ag_2_SO_4_), potassium dichromate (K_2_Cr_2_O_7_), sodium acetate trihydrate (CH_3_COONa·3H_2_O), ammonium chloride (NH_4_Cl), dipotassium hydrogen-phosphate (K_2_HPO_4_·3H_2_O), tert-butanol (TBA, 0.5 mmol/L), and ascorbic acid (LAA, 1 mmol/L).

Activated sludge was obtained from Ximingjiang Wastewater Treatment Plant, Nanning, Guangxi. WRF were purchased from the Guangdong Province Microbial Strain Preservation Center. Paper wastewater came from an OCC pulp and paper mill in Dongguan, Guangdong Province. The characteristics and DCS-related indicators are shown in Table 1.

#### 2.1.1. Sponge Cube and Photocatalyst Coating Procedure

The photocatalysts were made of titanium dioxide-coated sponge composite carriers as 1 cm^3^ cubes (98% porosity, 1.01–1.02 g/cm^3^ wet density, 422.2% water absorption, and 100% retention), with an average specific surface area of 4000 m^2^/m^3^. We described the method for the preparation of the titanium dioxide-coated sponge composite carriers in a previous paper [12]. The average particle size of titanium dioxide is 15 nm. Titanium dioxide was fixed on the surface of the sponge carrier, and a layer of TiO_2_ film was formed on the surface of the sponge carrier.

Polyurethane (PU) sponges serve a crucial role as reactors for chemical and biological treatment processes in wastewater management. Their distinctive porous structure offers an expansive surface area for microbial colonization, thereby enhancing the efficiency of pollutant–microbe interactions. Moreover, the chemical stability and mechanical robustness of PU sponges enable them to withstand harsh industrial conditions while maintaining their structural integrity, providing a solid physical foundation for sustainable wastewater treatment.

#### 2.1.2. Biofilm Culture

Biofilm cultivation is mainly divided into two parts, which are activated sludge and white rot fungi cultivation. The biofilm carrier utilized was a porous, honeycomb-structured polyurethane cube with a side length of 10 mm. The sponge carrier was cultured in a well-mixed internal loop airlift-driven reactor as detailed in our previous work [13].

In this experiment, intermittent culture was adopted to cultivate and domesticate the activated sludge with a hydraulic retention time (HRT) of 24 h. To adapt the activated sludge to the culture environment in the laboratory, the activated sludge was first cultivated for 2 days using a nutrient solution. The synthetic nutrient solution used for the activated sludge culture contained CaCl_2_ (4 mg/L), NH_4_Cl (54 mg/L), CH_3_COONa·3H_2_O (214 mg/L), NaH_2_PO_4_·2H_2_O (18 mg/L), and K_2_HPO_4_·3H_2_O (15 mg/L), and by using such an intermittent culture method, we could ensure the adequate propagation and cultivation of the activated sludge for subsequent experimental studies [14]. The activated sludge was domesticated using OCC paper wastewater and ozone photocatalytic post-effluent.

WRF were cultivated using a liquid potato medium. Under the conditions of 140 rpm and a temperature of 30 °C, the culture was incubated in a constant temperature oscillator for 5–6 days, and a high-quality mycelial liquid was prepared through the steps. After that, the ozone-catalytic effluent was used to domesticate the white rot fungi and the activated sludge-mixed white rot fungi system.

During the domestication, COD was detected every 12 h, and SVI and extracellular polymer content were detected when the COD removal rate stabilized. At the end of the domestication, the system was put into a polyurethane sponge carrier for coating for about 15 days.

#### 2.1.3. Experimental Procedure

**Figure 1:**
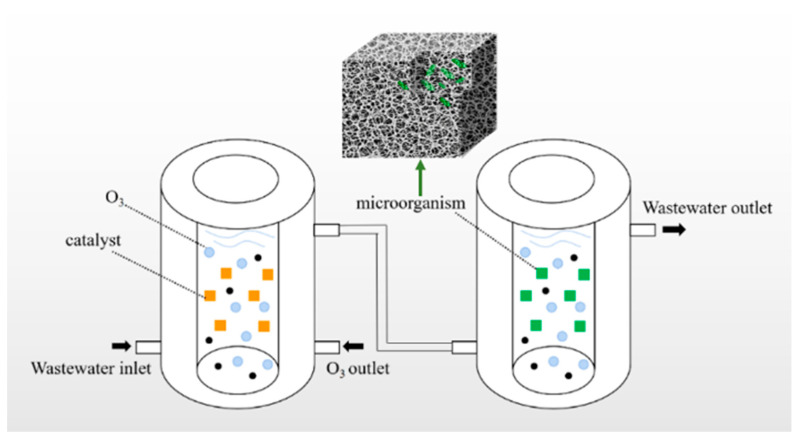
Experimental reactor.

Figure 1 shows the experimental reactor of this paper. First, the OCC pulping wastewater was pre-treated using ozone catalysis for 8 min, and the detailed steps are shown in the previous laboratory article [15]. The ozone-catalyzed water was removed into a 6500 mL beaker and set aside. Three groups of control tests were set up to degrade the water using polyurethane sponges loaded with activated sludge, WRF, and a mixture of the two microorganisms and to observe the degradation effects of the three groups of microorganisms on the DCS-related indices and COD. The experimental conditions were a 9% polyurethane carrier filling rate, an aeration volume of 2.5 L/min, and a running time of 12 h.

In addition, the OCC pulping wastewater was degraded by the microbial (B) system, which was compared with the ozone-catalyzed coupling biological process (OCB).

### 2.2. Analytical Determinations

In accordance with the stipulations of the GB 3544-2008 standard, which governs the discharge of water pollutants in the pulp and paper sector, an array of water quality indices was meticulously quantified. These encompassed assessments of pH, color intensity, biochemical oxygen demand after five days (BOD_5_), chemical oxygen demand (COD), ammonium ions (${NH}_{4}^{+}$), total nitrogen (TN), and total phosphorus (TP). The acidity or alkalinity of the water samples, denoted by pH, was ascertained utilizing a standard pH meter. The measurement of each parameter was strictly carried out in accordance with the regulations specified in their respective standard method guidelines, that is, in accordance with the national standard methods of the People’s Republic of China. Specifically, the COD was measured according to HJ 828-2017, BOD_5_ according to HJ 505-2009, total nitrogen in accordance with HJ 636-2012, ammonia nitrogen following the procedures outlined in HJ 535-2009, total phosphorus as per GB11893-89, and color intensity based on the criteria established by HJ 1182-2021. In this case, BOD_5_ and COD respond to the content of organic matter in the wastewater, and as the value decreases, the organic matter content also decreases.

The DCS-related characters were measured, and the zeta potential and average particle size were determined by a Nano-ZS90X nano-particle size analyzer. The water samples were centrifuged at 2000 r/min for 30 min. The supernatant samples were dried at 105 °C until the mass was constant and then weighed as the DCS content of the treated water samples.

To scrutinize the spatial distribution of the biofilm on the carrier’s surface at both the pre- and post-reaction stages, specimens were subjected to analysis using a high-resolution scanning electron microscope (SEM, FEI-NOVA NANO 230). For those samples that were inhabited by microorganisms, a pretreatment process was applied, which was previously defined by the methodologies of Li et al. [16].

The assessment of microbial activity throughout the experimental period was facilitated by employing confocal laser scanning microscopy (CLSM). This advanced technique enabled the differentiation and examination of the distribution patterns of both viable and non-viable bacterial populations within the biofilm matrix. For this purpose, a live/dead bacterial staining kit (BBcellprobe N01/PI, Shanghai Besbio Biomedical Technology Co., Ltd., Shanghai, China) was utilized, followed by microscopic observation under a Leica TCSSP8 instrument (Leica, Wetzlar, Germany). This process yielded fluorescence imagery of the biofilm at a magnification of 100×.

In order to elucidate the shifts in the composition of the microbial community over the course of the experiment, sponge samples were cryopreserved post the completion of the stand-alone biological reactions. Subsequently, Shanghai Meiji Biomedical Technology Co., Ltd. (Shanghai, China) was engaged to perform an in-depth interactive analysis aimed at characterizing the environmental microbial diversity within the samples.

## 3. Results and Discussion

### 3.1. Effects of the Combination Strategy on OCC Pulping Wastewater Treatment

#### 3.1.1. Comparison of Degradation Performance of Ozone-Catalyzed Oxidized Effluent by Different Microbial Systems

In the OCB system, the selection of microbial populations affects the degradation of the ozone-catalyzed effluent because of the different biocompatibilities of microbial populations to polyurethane sponges. Therefore, three different microbial communities, i.e., activated sludge, WRF, and a mixed system of activated sludge and WRF, were selected to compare their degradation effects on ozone-catalyzed oxidation of effluent water in order to select a suitable microbial system. Meanwhile, the zeta potential and average particle size can describe the DCS content to some extent, so the changes in COD, zeta potential, and average particle size were investigated.

**Figure 2:**
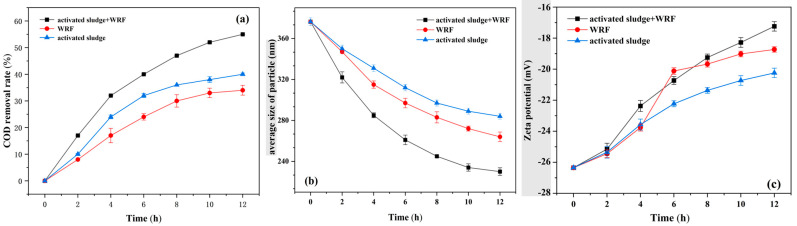
The effect of different microbial systems on the removal efficiency of COD (**a**), average particle size (**b**), and zeta potential (**c**) in the ozone-catalytic oxidation effluent.

Figure 2a compares the effect of different microbial populations on COD removal from the ozone-catalytic oxidation effluent. After 12 h of reaction, the degradation rate of COD by mixed microbial populations was 55%, which was higher than that of activated sludge alone and WRF alone (39% and 31%), indicating that the mixed microbial populations were more biocompatible with the polyurethane sponge and more adaptable to the ozone-catalytic oxidation of effluent with better degradation ability. The degradation effect of WRF alone on the ozone-catalyzed oxidation effluent was generally due to the poor adaptability and weak shock resistance of single strains. In the mixed microbial population, the degradation of wastewater was greatly improved by the activated sludge, which first degraded the macromolecules in the ozone-catalyzed oxidized effluent, metabolically produced acids to regulate the pH value of the effluent, and enhanced the activity of laccase produced by the white rot fungi, which resulted in the degradation of fatty acids and lignin.

Figure 2b shows the effect of different microbial populations on the average particle size of colloids in the ozone-catalyzed oxidized effluent. In the mixed microbial population, the average particle size of colloids was reduced by 38%, which was better than that of activated sludge alone and white rot bacteria. The reduction in particle size could be attributed to the degradation of lignin by lignin-degrading enzymes, which release charged particles attached to the surface of the paper fibers, thus reducing the average particle size of the colloids. The effect of different microbial populations on the zeta potential of OCC wastewater was evident, as shown in Figure 2c. The rate of increase in the zeta potential of the white rot bacteria alone decreased after 6 h of the reaction, which is consistent with the slowing down of COD degradation after 6 h in Figure 2a. In general, the increase in zeta potential can be attributed to two factors as follows: some organic molecules in the water are digested and metabolized by the microorganisms as a nutrient source, and the microorganisms produce a large number of proteins during the growth process. It has been shown that these proteins are positively charged and, therefore, the absolute value of the zeta potential decreases, making it easier for the DCSs in the water to flocculate and settle. Therefore, WRF mixed with activated sludge was chosen as a hybrid microbial system for OCB and B.

#### 3.1.2. Optimization of Microbial System

After deciding to use the mixed microbial system, the operation of the microbial system was optimized using a response surface approach by the software Design-Expert 8.0. The following four factors were examined: pH, aeration, filling rate, and reaction time. A list of experimental factors and level design is shown in Table 2.

**Figure 3:**
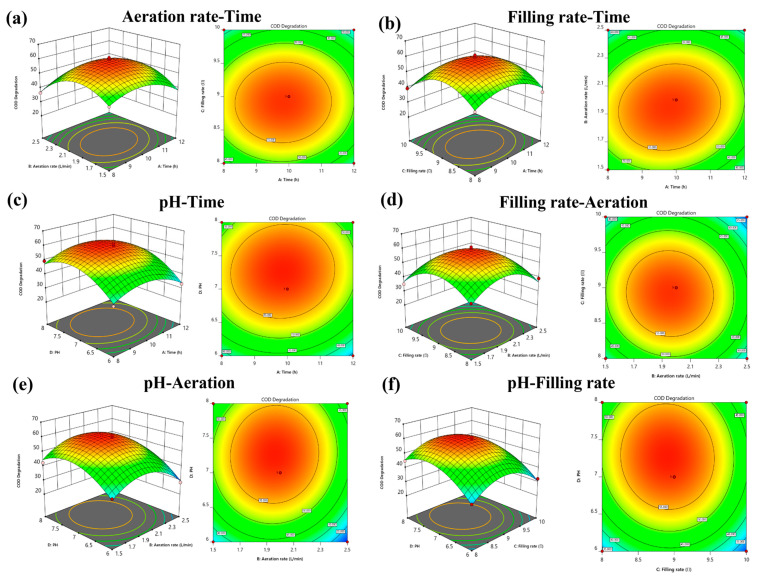
COD removal rate as response value response surface diagrams and diagrams: (**a**)Aeration-Time; (**b**) Filling rate-Time; (**c**) pH-Time; (**d**) Filling rate-Aeration; (**e**) pH-Aeration; and (**f**) pH-Filling rate. The colours in the graph from red to green indicate a gradual decrease in the rate of degradation.

Figure 3 shows the response surface and contour plots generated based on the regression model. It can be seen that all four influencing factors have an effect on the microorganisms, while a certain synergistic mechanism exists. In Figure 3c,e,f, it can be seen that the COD degradation efficiency of the mixed microbial system in ozone-catalyzed water first increased and then decreased with the increase of initial pH. The highest COD degradation efficiency was observed at an initial pH of 7. The contour lines on the alkaline side were sparse compared with the acidic side, indicating that the acidic condition had more influence than the alkaline condition. Figure 3a,d,e show that the aeration rate has an effect on the degradation efficiency of COD in OCC pulping wastewater in terms of the interaction of different factors. The highest removal efficiency was achieved when the aeration rate was 2.5 L/min. If the aeration rate is too large, it may cause the mixed microorganisms to be dislodged from the inside of the polyurethane sponge. Figure 3b,d,f show that the removal efficiency of COD in OCC pulping wastewater first increased and then decreased with an increase in the filling rate, and the highest degradation efficiency was achieved at a filling rate of 9%. The reaction duration also had an effect on the degradation of COD. Mixed microorganisms can produce enough free radicals to rapidly degrade organic matter within a certain reaction time. At the same time, mixed microorganisms can produce specific microbial enzymes, such as laccase, which have decomposition effects on some specific DCS components in OCC pulping wastewater. Overall, compared with the reaction time, pH, aeration, and the filling rate had a more significant effect on the COD removal rate. Under the optimal conditions, the highest COD removal for ozone-catalyzed water was 60.95%. The optimal operating conditions for microorganisms in the OCB were determined by response surface calculation to be a pH of 7, a filling rate of 9%, an aeration volume of 2 L/min, and a reaction time of 10 h.

### 3.2. Degradation Effect of OCB and B for OCC Pulping Wastewater

**Figure 4:**
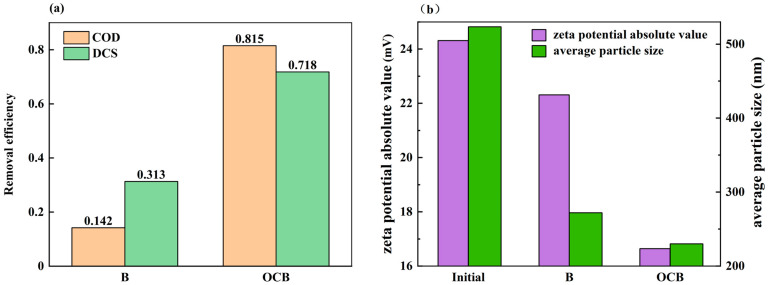
Character changes after degradation by OCB and B: DCS, COD (**a**) and zeta potential, average particle size (**b**).

Figure 4 shows the changes in zeta potential, average particle size, and DCS solid content of OCC paper wastewater after degradation by OCB and B. These three parameters can indicate the overall changes in DCSs in OCC pulping wastewater. After treatment by the OCB system, the DCS solid content removal reached 71.8%, the absolute value of the zeta potential decreased from 24.31 mV to 16.64 mV, and the average particle size decreased from 523.4 nm to 218.2 nm, which is a significant improvement in the removal of mucilage from the wastewater when compared with the B system (31.2%, 22.31 mV, and 275 nm). Meanwhile, the removal of COD reached 81.5%, which was higher than that of system B (33%), indicating that OCC pulping wastewater has better biodegradability after ozone-catalyzed oxidation, with more vigorous growth of microorganisms and higher content of proteins in the extracellular polymers, which leads to destabilization of negatively charged colloids in the water and coalescence of the colloids [17]. Interestingly, OCC pulping wastewater treated by ozone-catalyzed oxidation showed an elevated absolute zeta potential of 26.35 mV. It has been shown [18] that ozone-catalyzed oxidation degrades microfibers, which results in the re-release of the anions attached to them into the system, causing a decrease in the zeta potential. At the same time, the average particle size was reduced to 371.1 nm, which was helpful for the microbial subsequent treatment. At the same time, both the OCB system and B system had greater removal of average particle size, which is mainly because the fibers in paper wastewater contain lignin components, which are easily degraded by microbial dominant strains, such as white rot fungi, resulting in fiber fragmentation and particle size reduction.

Based on the analysis of the above data, ozone-catalyzed oxidation and microorganisms possess good synergistic effects.

### 3.3. OCB Degradation Mechanism

**Figure 5:**
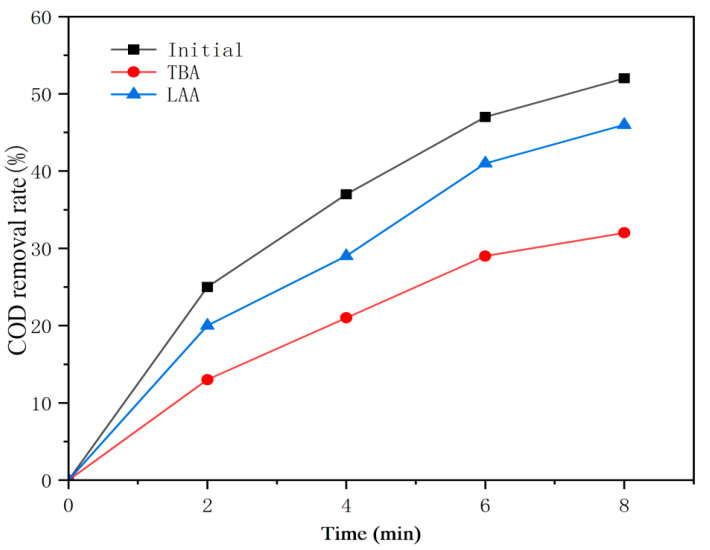
Free radical capture experiment (the experimental results are averaged over three times).

The reaction mechanism during ozone catalysis in OCB was verified using a free radical trapping agent. As shown in Figure 5, the removal of COD from OCC paper wastewater was reduced from 52% to 45% and 31% by using two free radical scavengers, i.e., tert-butanol (TBA, 0.5 mmol/L) and ascorbic acid (LAA, 1 mmol/L), which capture ${\cdot O}_{2}^{-}$ and ·OH, respectively. This indicates that in the OCB system, both ${\cdot O}_{2}^{-}$ and ·OH were produced, and ·OH was dominant. The activated oxygens attack the organics, which are difficult to degrade in the wastewater, forming intermediates that are subsequently mineralized in biodegradation.

### 3.4. Microbial Response

#### 3.4.1. SEM Analysis

**Figure 6:**
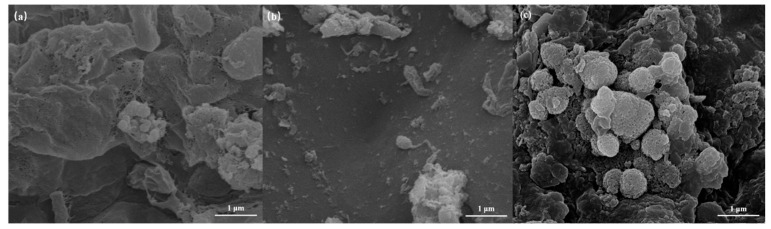
SEM of the biosponge carrier: (**a**) initial; (**b**) after the B reaction; (**c**) after the OCB reaction.

Figure 6 represents the microscopic morphology of microorganisms within the polyurethane sponge. Figure 6b shows the real water biofilm map after microbial treatment only, indicating that under the influence of high organic load, microbial microorganisms could not adapt to the higher pollution load and inactivated and gradually died. Figure 6a shows a mixed microbial system loaded on a polyurethane sponge prior to the reaction. The microbial growth was good with many microorganisms growing on the surface of the carrier. Figure 6c shows the carrier and microorganisms after the reaction of the ozone-catalyzed oxidation microbial system, indicating that there is still intact biofilm growth inside the carrier, compared with Figure 6a. The biofilm growth is better, and the dominant bacterial species has been identified.

**Figure 7:**
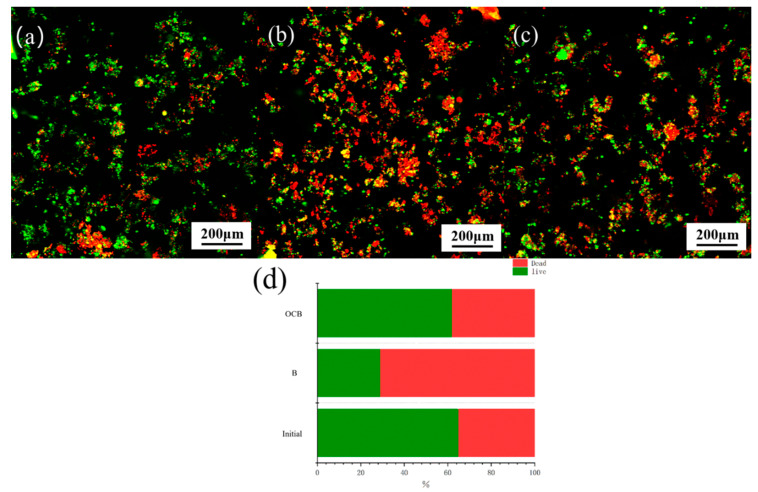
The distribution of active and dead bacteria before and after degradation: (**a**) before degradation; (**b**) after B degradation; (**c**) after OCB degradation; and (**d**) the ratio of active and dead cells after B and OCB degradation.

Figure 7 shows biofilm images before and after the treatment of OCC pulping wastewater by the B treatment and OCB system as observed by CLSM. Figure 7a–c show the staining results of live and dead bacteria. When the microorganisms were used to directly treat the OCC pulping wastewater (Figure 7b), a large number of microorganisms died, which indicated that the OCC pulping wastewater had poor biodegradability, and the microorganisms had difficulty in degrading it directly, which coincided with the results of the increase in the number of dead bacteria (71%) in Figure 7d. After the reaction of the OCB system, the polyurethane carrier-loaded microorganisms still maintained good activity, and the survival rate (62%) was close to that of the original microorganisms. The polyurethane sponge provided stable reaction conditions for the microorganisms and provided some protection for the microorganisms, so the microorganisms had the ability to resist the impact of the high organic loads. Microorganisms can use these intermediates as carbon sources for their own growth. In addition, combined with Figure 6c, it was observed that after 10 h of biodegradation, although the microorganisms showed some decay, new dominant strains were screened, which may enhance the treatment of OCC pulping wastewater in the future.

#### 3.4.2. Microbial Diversity Analysis

**Figure 8:**
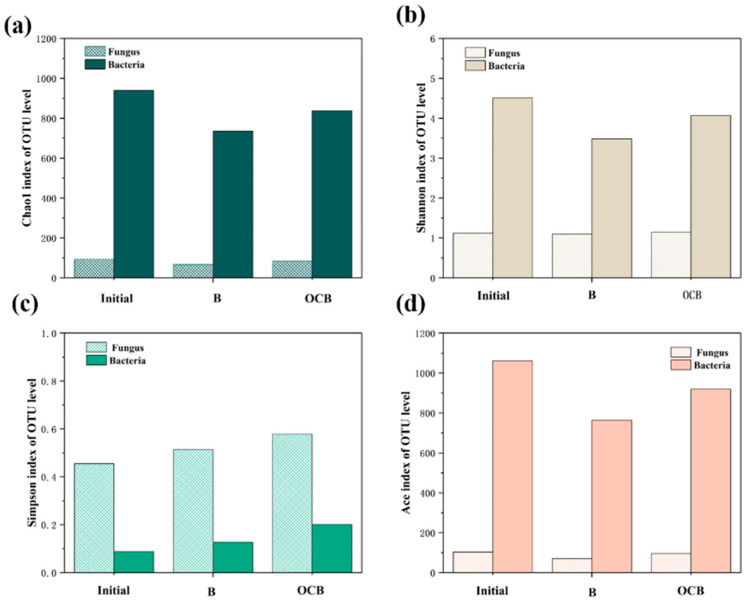
Microbial diversity index analysis at OTU level. (**a**) Chao1 index; (**b**) Shannon index; (**c**) Simpson index; (**d**) Ace index.

Alpha diversity is used to assess the abundance of species within a certain range or system, including the Ace index, Chao1 index, Shannon index, and Simpson index. the Ace index and Chao1 index are used to assess the species richness in a system [19]. As can be seen from Figure 8a,d, the Ace and Chao1 indices of the biofilm samples treated by the OCB and B systems were lower than those of the initial samples, which indicated a significant decrease in the species richness. This is mainly due to the toxic effect of the organic matter in the OCC pulping wastewater, where some microorganisms could not survive under the higher pollution load. In addition, there is a natural selection process among microbial communities under this organic load [20]. It should be emphasized that in the OCB system, the Ace and Chao1 indices decreased to relatively low values. This is due to the fact that after ozone catalysis, the macromolecular organic matter in the OCC pulping wastewater is converted into intermediates with relatively low toxicity. The Shannon and Simpson (Figure 8b,c) indices were used to assess the microbial diversity in the systems. Compared with the original samples, the Shannon and Simpson indices of both the B and OCB systems decreased, indicating that the microbial diversity also varied with the growth environment. The diversity of microbial communities also decreased to different degrees after the treatment of OCC pulping wastewater. In addition, the Shannon index of fungi remained relatively stable, which may be due to the presence of dominant fungal species and the fact that certain fungi can maintain their growth level by degrading polyurethane sponges [21].

**Figure 9:**
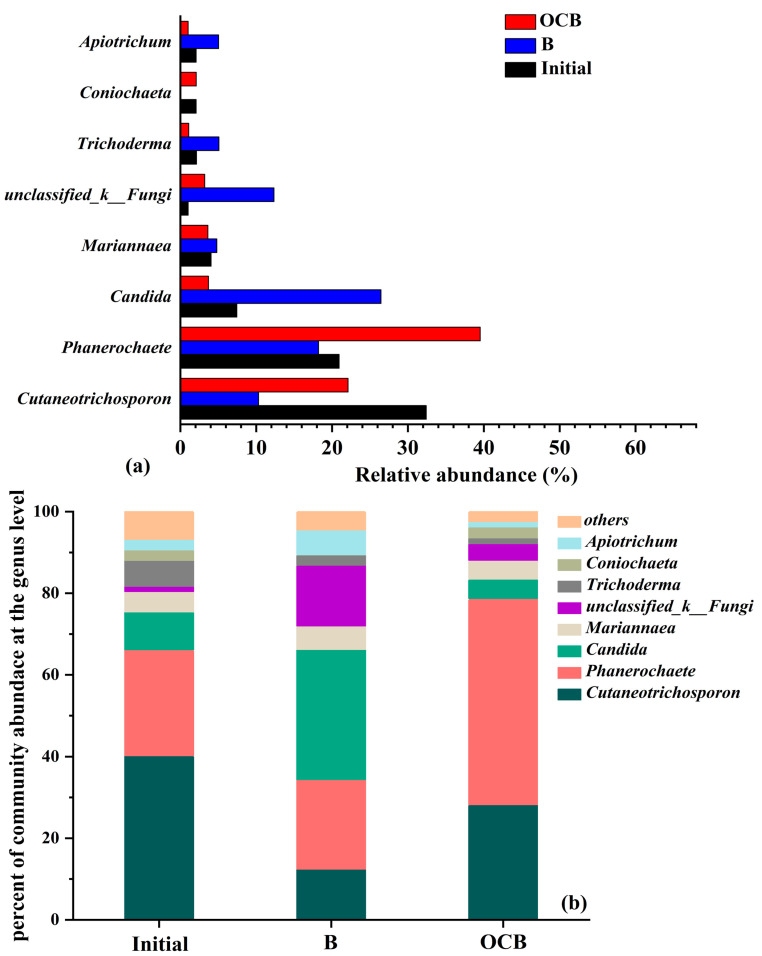
Analysis of significant differences in fungal community changes (**a**) and changes at genus-level diversity (**b**) before and after B and OCB system degradation.

To further investigate the functional fungal species in the mixed microbial community, Figure 9a shows the changes in fungal genus-level diversity in three different samples of biofilms. Figure 9b presents the analysis of significant differences in community changes.

In Figure 9a, the original colonies mainly consisted of fungi such as *Cutaneotrichosporon*, *Phanerochaete*, *Candida*, *Mariannaea*, *Coniochaeta*, and *Trichoderma*. After reactions in the different systems, there were significant differences in the abundance of these fungal species. It can be observed that under the impact of high organic load in OCC pulping wastewater, the fungal genera underwent significant selection and differentiation. *Cutaneotrichosporon* is capable of degrading hydrolysis products during the degradation of lignocellulose [22], which may be the reason for its higher survival rate in the OCB system compared with the B system, as shown in Figure 9b. *Phanerochaete* is a WRF that has good degradation effects on lignin and fatty acids in papermaking wastewater [23]. After the ozone-catalyzed oxidation decomposition of OCC pulping wastewater, the survival rate of *Phanerochaete* in the microbial system was effectively improved, as shown in Figure 9b. *Candida* is a yeast genus that has a unique promoting effect on alcohol and acid degradation. It had a higher abundance in the B system, but its degradation effect on OCC pulping wastewater was not significant, as mentioned earlier. Additionally, *Coniochaeta* is also commonly used for the degradation of lignocellulose [24]. In the initial sample, the dominant fungus was white rot fungus (*Phanerochaete*), indicating that after the introduction of white rot fungus during the cultivation and domestication process, it had good compatibility with the activated sludge, and the community gradually grew stronger during the domestication process. In the OCB system, the abundance of white-rot fungus was higher, which also indicates that ozone-catalyzed oxidation helps enhance the activity of dominant fungal species. Ozone-catalyzed oxidation and the microbial system have a good synergistic effect.

After treatment with both systems, new bacterial genera appeared in OCC pulping wastewater. *Candidatus_Berkiella* and *Nocardia* were the new dominant genera in the OCB samples. This community change is mainly due to the presence of amides and their derivatives, lignin, fatty acids, and other organic substances in OCC pulping wastewater. In the B samples, bacterial genera with the ability to degrade such substances have a competitive advantage. In the OCB system, ozone helps the growth of microorganisms that can utilize intermediate products as their own nutrients. This is also the reason why the efficiency of OCC pulping wastewater degradation is higher in the synergistic system than in the B system. Finally, it has been reported that *Pseudomonas* has a certain degradation function for polyurethane sponges [25], with a large proportion in both the B and OCB samples.

## 4. Conclusions

In this study, an effective combined advanced oxidation-biological (OCB) process was proposed for OCC wastewater. After an 8-min ozone-catalyzed reaction, a polyurethane sponge loaded with a mixture of activated sludge and WRF was put into the system, and the degradation efficiencies of the OCB system for COD and BOD_5_ reached 81.5% and 85.1%, respectively, with a significant reduction in the DCS-related indices, under the reaction conditions of a pH of 7, a filling rate of 9%, an aeration rate of 2 L/min, and a reaction time of 10 h. The OCB system was also effective in the removal of COD and DCS, and the degradation performance of OCC wastewater was good. The OCB system showed good performance in COD degradation and effective removal of DCS. The microbial diversity test showed that the reaction process would optimize the screening of suitable strains for the degradation of OCC pulping wastewater, such as the fungi *Phanerochaete* and *Pseudomonas*, which can degrade polyurethane sponges to a certain extent. Ozone-catalyzed oxidation could help to improve the degradation performance of the mixed microbial community, which indicated that ozone oxidation and microbial activity had a synergistic effect. Moreover, the enormous specific surface area of polyurethane sponges provides a good and relatively stable condition for the immobilization of microorganisms, allowing them to be less affected by the shear forces of water flow. This facilitates their fixation on the polyurethane sponge, thereby enabling biological metabolism. This demonstrates the feasibility of introducing WRF into the ozone-catalyzed oxidation-coupled biodegradation of difficult-to-degrade organic compounds and DCSs in OCC pulping wastewater.

## Figures and Tables

**Table 1:** Characterization of OCC wastewater.

Parameter	Real Wastewater	After Ozone Catalyst	After the Ozone-Catalyzed Coupling Biological Process
COD (mg/L)	953	453	176
BOD_5_ (mg/L)	254	210	36
NH_4_^+^ (mg/L)	24.23	25.03	7.3
TN (mg/L)	13.55	11.97	9.2
TP (mg/L)	0.61	0.67	0.67
SS (mg/L)	2439	1749	534
pH	7.8	7.45	7.2
Chromaticity	1789	153	114
Zeta potential (mV)	−24.31	−26.35	−16.64
DCS solid content (g/L)	1.63	1.33	0.46
Average particle size (nm)	523.4	371.1	218

**Table 2:** Response surface parameter table.

Level	Factor			
	A Time/h	B Aeration Rate/Min·L	C Filling Rate/%	D pH
−1	8	1.5	9	6
0	10	2	10	7
1	12	2.5	11	8

## Data Availability

The data are contained within this article.
